# Fracture Strength of CAD/CAM Endocrown and Post-Core Restorations with Fiber Strip Reinforcement in Mandibular Premolars

**DOI:** 10.3390/jfb17050248

**Published:** 2026-05-17

**Authors:** Kerem Yılmaz, Hakan Aydın, Zeynep Soylu, Özge Çiloğlu, Esma Fatıma Delican, Mehmet Mustafa Özarslan, Fehmi Gönüldaş

**Affiliations:** 1 Department of Prosthodontics, Faculty of Dentistry, Antalya Bilim University, 07190 Antalya, Turkey; 2 BilimDent Oral and Dental Health Center, Antalya Bilim University, 07190 Antalya, Turkey; 3 Department of Endodontics, Faculty of Dentistry, Antalya Bilim University, 07190 Antalya, Turkey; 4 Department of Prosthodontics, Faculty of Dentistry, Ankara University, 06560 Ankara, Turkey

**Keywords:** resin-nanoceramic, fiber strip reinforcement, polymer-based dental materials, endocrown, fracture strength, mandibular premolar

## Abstract

This study evaluated the effects of restorative material, restoration type, and fiber strip reinforcement on the fracture strength (FS) of endocrown (EC) and post-core (PC) restorations in endodontically treated premolars. Specimens were allocated according to restorative material [resin-nanoceramic (RNC) or feldspathic ceramic (FC)], restoration type (EC or PC), and reinforcement [fiber strip-reinforced (FR) or -non-reinforced (NF)]. FS was determined using a universal testing machine under axial loading. Statistical analysis was performed using three-way ANOVA and Bonferroni tests (α = 0.05). Material, restoration type, and reinforcement significantly affected FS (*p* < 0.05). RNC restorations exhibited higher FS than FC restorations (861 ± 181 N vs. 715 ± 212 N; *p* < 0.001). EC restorations exhibited higher FS than PC restorations (828 ± 173 N vs. 748 ± 236 N; *p* = 0.046). FR groups exhibited higher FS than NF groups (848 ± 180 N vs. 728 ± 222 N; *p* = 0.003). The highest FS was observed in the RNC–PC–FR group (965 ± 144 N), whereas the lowest occurred in the FC–PC–NF group (480 ± 177 N). Although EC restorations showed higher FS than PC restorations, the effect of restoration type depended on material and reinforcement.

## 1. Introduction

The long-term restorative success of endodontically treated teeth (ETTs) with extensive coronal tissue loss is compromised by the loss of structural integrity and associated biomechanical weakness. For many years, post-core (PC) restorations have been one of the most commonly used approaches for rehabilitating these teeth. This approach involves anchoring a core substructure to the root via a post placed in the root canal, followed by restoration with a crown [1]. However, Zuli et al. [2] and Singh et al. [3] reported that post space preparation may increase the risk of fracture by removing radicular dentin, reducing the remaining dentin thickness, and creating microdefects in the root dentin.

Advances in adhesive dentistry and computer-aided design and computer-aided manufacturing (CAD/CAM) systems and materials have enabled the development of more conservative approaches for the restoration of ETTs. In this context, the endocrown (EC) concept, first defined in 1999, has enabled the fabrication of monolithic CAD/CAM restorations that do not require post placement, owing to the macroretention provided by the pulp chamber and the adhesive bonding properties of resin cements [4]. Today, ECs have become an important treatment option for premolar and molar teeth, as they can be designed and applied in a single session and reduce the need for temporary restorations [5]. Previous studies [6,7] have reported that the retention of ECs is enhanced by their central mass extending into the pulp chamber. This design may also influence biomechanical behavior and fracture strength (FS) by altering stress distribution within the restoration–tooth complex.

The amount of residual coronal dentin is considered one of the most important factors affecting FS in ETTs; an increase in this amount is generally associated with higher FS values [8]. This finding highlights the importance of preserving the remaining tooth structure to maintain the biomechanical integrity of the restoration–tooth complex. In addition, other major factors affecting FS in PC and EC restorations include the type of CAD/CAM restorative material used (resin-based and ceramic materials) [9,10], restoration type (PC or EC) [11,12], preparation design [13], and base–core material [14,15].

Recent developments in dental restorative materials have focused on improving resin matrix composition, filler characteristics, and filler–matrix coupling to enhance mechanical and functional performance [16]. In this context, ceramics and resin-containing CAD/CAM materials are commonly used in restorative dentistry [17]. Feldspathic ceramics (FCs) are widely used because of their optical properties, which resemble those of natural teeth [18]. However, their high inorganic phase content and absence of a polymer phase make them more susceptible to crack initiation and propagation under sudden loading [19]. Their high elastic modulus (E) may also promote stress concentration at the adhesive interface and cavity corners, resulting in a more rigid biomechanical response than dentin [20]. In contrast, resin-nanoceramics (RNCs) have a hybrid structure composed of a polymer matrix and ceramic fillers, with lower E values closer to dentin, which may contribute to partial absorption of occlusal forces and more homogeneous stress distribution [21].

In hybrid CAD/CAM materials with a polymer matrix–ceramic filler structure, microstructural heterogeneity may activate energy-dissipating mechanisms such as crack deflection and crack bridging, which may shift fracture behavior toward more repairable fracture patterns (FPs) [13]. Hassouneh et al. [22], Uzun et al. [23], and Yılmaz et al. [24] reported that irreparable FPs are less frequent in RNCs than in FCs. Consistently, Chen et al. [25] reported significantly lower E values for RNC blocks than for glass-ceramics. However, FS findings remain inconsistent; Hassouneh et al. [22] reported lower FS values for RNC than for lithium disilicate in premolar endocrowns, despite more repairable FPs, whereas Alnajjar et al. [26] reported adequate FS for RNC crowns after thermomechanical aging, together with a higher proportion of repairable FPs.

These studies indicate that the biomechanical behavior of the restoration–tooth complex should be evaluated not only in terms of the mechanical properties of the material used, but also in conjunction with factors such as restoration type, cavity geometry, and base–core structure. The effect of base–core structure on the biomechanical behavior of the restoration–tooth complex is not yet fully understood, particularly considering the limited residual dentin volume and the potential for high bending moments in premolar teeth. However, only a limited number of studies have investigated the FS and FP of EC and PC restorations fabricated from different CAD/CAM materials, particularly in premolar teeth, where biomechanical conditions differ from those in molars.

In addition to restorative materials and restoration type, various strategies have been proposed to modify the mechanical behavior of the base or core structure of restorations. Fiber reinforcement techniques, particularly fiber strip applications, have attracted attention as methods with the potential to influence load transfer within the restoration–tooth complex. The placement of fiber strips as an intermediate layer within the resin matrix may contribute to stress absorption and redistribution, and this effect has been reported to depend on the position of the fiber strip and its interaction with the surrounding matrix [27].

Resin composite (RC)-based materials are widely used as bases in ECs and as core materials in PCs; however, their biomechanical contribution in these restorations remains inconsistent. A three-dimensional finite element analysis study [14] reported that a base layer could provide a force-buffering effect in ECs by promoting more homogeneous stress transfer because of its low E value. Conversely, another study [28] found that fiber reinforcement of the base layer did not significantly affect FS. These conflicting findings indicate that the role of base–core modification, particularly fiber strip reinforcement, has not been fully clarified. This gap is especially relevant for premolar teeth, where limited residual dentin volume and different load-transfer pathways may influence FS and FP. Therefore, further investigation is needed to evaluate the combined effects of restoration type, restorative material, and fiber strip reinforcement in a standardized premolar model.

In recent years, it has been recognized that the performance of restorative systems depends not only on material selection but also on the interaction between material properties, structural design, and preparation strategies [29]. However, it remains unclear whether fiber strip reinforcement produces comparable effects when applied as a base modification in ECs or as a core modification in PCs, and whether this effect varies between FC and RNC materials. To the best of the authors’ knowledge, no previous in vitro study has simultaneously evaluated the combined effects of restorative material, restoration type, and fiber strip reinforcement on the FS and FPs of CAD/CAM EC and PC restorations in mandibular premolars. Accordingly, the aim of the present study was to evaluate the effects of fiber strip reinforcement applied to the base or core structure on the FS and FPs of EC and PC restorations fabricated from FC and RNC CAD/CAM materials in endodontically treated mandibular premolars. The null hypothesis was that restorative material, restoration type, and the presence of fiber strips in the base/core structures would not have a significant effect on FS.

## 2. Materials and Methods

### 2.1. Selection and Grouping of Specimens

This study was designed as an in vitro experimental study conducted on extracted human teeth. Before starting the study, approval was obtained from the Antalya Bilim University Health Sciences Research Ethics Committee (Decision No: 2025/072; approval date: 18 March 2025). Specimens were selected from mandibular premolars extracted due to periodontal or orthodontic indications at the Antalya Bilim University BilimDent Oral and Dental Health Application and Research Center clinic. Mandibular premolars were selected to provide a standardized premolar model with limited residual dentin volume and clinically relevant biomechanical susceptibility after endodontic treatment. All specimens were examined macroscopically and radiographically by the researchers before being included in the study and evaluated according to the inclusion and exclusion criteria.

Inclusion criteria were defined as mandibular premolars with similar dimensions [mesiodistal: 6.4 ± 0.5 mm, buccolingual: 7.9 ± 0.5 mm], a coronal height of 7 ± 1 mm, and a root length of 14 ± 1 mm; a single root canal, caries-free, vital, with complete root development and preserved morphological integrity. These dimensional measurements were performed using a digital caliper in all specimens. Exclusion criteria were defined as specimens that had previously undergone endodontic treatment, restorative procedures, cracks or fractures in the root or crown, pathological resorption, multiple canals or morphological variations, and teeth other than mandibular premolars.

The collected specimens were stored in a 0.1% thymol solution at 4 °C until the start of the study to prevent microbial contamination. Specimens stored for longer than one month were excluded from the study due to the risk of microstructural changes.

**Figure 1:**
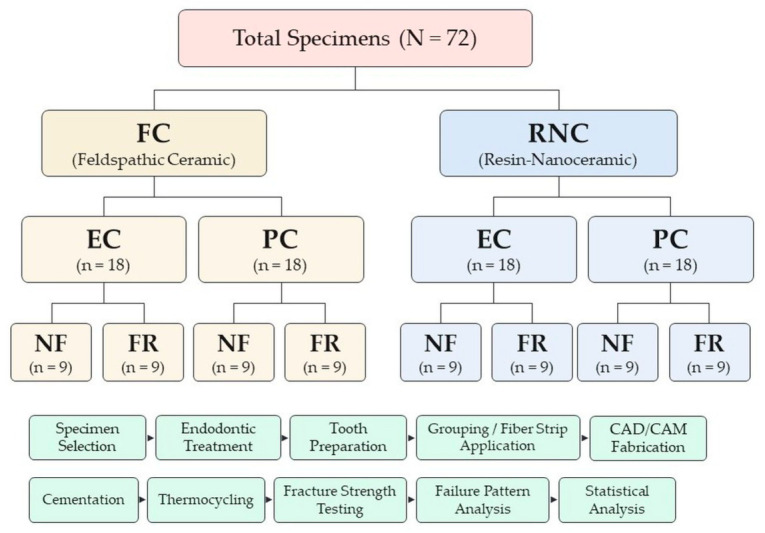
Experimental grouping of specimens and study workflow. EC, endocrown; PC, post-core; NF, fiber strip-non-reinforced; FR, fiber strip-reinforced.

Two main groups were formed according to the type of restorative material. The first group consisted of specimens prepared from FC blocks (Cerec Blocs C, Dentsply Sirona, Bensheim, Germany); the second group consisted of specimens prepared from RNC blocks (Cerasmart 270, GC Corp., Tokyo, Japan). Both main groups were subdivided into EC and PC subgroups according to the type of restoration. The subgroups were further divided into fiber strip-reinforced (FR) and non-reinforced (NF) groups using pre-impregnated bidirectional glass fiber (GF) strips (everStick NET, GC Europe, Leuven, Belgium) to form the final working groups. In the PC groups, a fiber post (Cytec HT Glassfiber, Hannenkratt, Königsbach-Stein, Germany) was used as the root canal post, and nanocomposite RC (Filtek Ultimate, 3M ESPE, Seefeld, Germany) was used as the core material (Figure 1).

The composition of the materials used in the study is presented in Table 1.

The sample size was determined by power analysis using G*Power software (ver. 3.1.9.7, Heinrich-Heine University, Düsseldorf, Germany). The significance level was set at 0.05, the statistical power at 0.90, and the effect size at f = 0.40. Based on these parameters and eight experimental groups, the minimum required total sample size was calculated as 68 specimens [8]. To ensure equal distribution among groups and to compensate for potential specimen loss, the total sample size was increased to 72 specimens (*n* = 9 per subgroup).

Randomization was performed using a computer-assisted random number generator after specimen eligibility assessment. The allocation sequence was generated before group assignment, and specimens were assigned to the experimental groups using coded labels. To reduce potential operator bias, experimental procedures were distributed among the researchers according to predefined roles, with each procedure performed consistently by the same researcher across the experimental groups. Complete operator blinding during specimen preparation and restoration fabrication was not feasible because the restoration type and fiber strip application were visually identifiable. However, the primary outcome, FS, was objectively recorded by the universal testing machine, and FP classification was performed according to stereomicroscopic examination and predefined fracture criteria.

### 2.2. Endodontic Preparation of Specimens

In all teeth, a horizontal section was made approximately 2 mm coronal to the enamel–cement junction (CEJ) using a water-cooled separating bur. This procedure was performed to equalize root lengths and create standardized restoration conditions. After sectioning, access cavities suitable for the pulp chamber were prepared using a high-speed handpiece (NSK M900L, NSK, Tochigi, Japan) and a diamond bur (No. 806, Dentsply Sirona, Ballaigues, Switzerland). The working length was determined using a K-type hand file advanced to the apical foramen.

Following apical patency assessment, root canals were shaped according to the manufacturer’s recommendations using a nickel–titanium rotary file system (ProTaper Universal, Dentsply Maillefer, Ballaigues, Switzerland) and an endodontic motor (X-Smart, Dentsply Maillefer, Ballaigues, Switzerland). Root canal preparation was completed up to the F5 file, 1 mm short of the apical foramen. Frequent irrigation with 5.25% sodium hypochlorite (NaOCl) was performed after each rotary instrument change to dissolve organic tissue and reduce the microbial load.

To remove the smear layer and expose the dentinal tubules, the canals were irrigated with 17% ethylenediaminetetraacetic acid (EDTA) for 1 min after preparation was completed. Following this step, a final rinse was performed with 5.25% NaOCl. The canals were then dried using sterile paper points.

Root canal filling was performed using the lateral condensation technique. Gutta-percha (GP) cones (ProTaper Next, Dentsply Sirona, Charlotte, NC, USA) were used as master cones. An epoxy resin-based root canal sealer (AH Plus, Dentsply DeTrey, Konstanz, Germany) was used as the root canal sealer.

After obturation, excess GP was removed from the root canal orifices using a heated plugger. Coronal sealing was achieved with glass ionomer cement (Vitrebond, 3M ESPE, St. Paul, MN, USA). All treated teeth were stored in an incubator at 37 °C (UM 400, Memmert GmbH, Schwabach, Germany) for 48 h to allow the root canal and coronal filling materials to fully set.

### 2.3. Tooth Embedding and EC Preparation

Teeth were embedded in polypropylene tubes with an inner diameter of 2.2 cm and a length of 3.5 cm using self-polymerizing acrylic resin (ProBase Cold, Ivoclar Vivadent, Schaan, Liechtenstein). During the embedding process, the CEJ was positioned approximately 1 mm above the acrylic resin surface. This configuration simulated the alveolar bone level and provided standardized conditions for all specimens.

The pulp chamber walls were smoothed, and undercuts were eliminated using a round-ended diamond bur. This procedure was performed to achieve a cavity geometry suitable for EC restorations. The axial wall inclination was standardized to 8–10° using a double-cone diamond bur.

**Figure 2:**
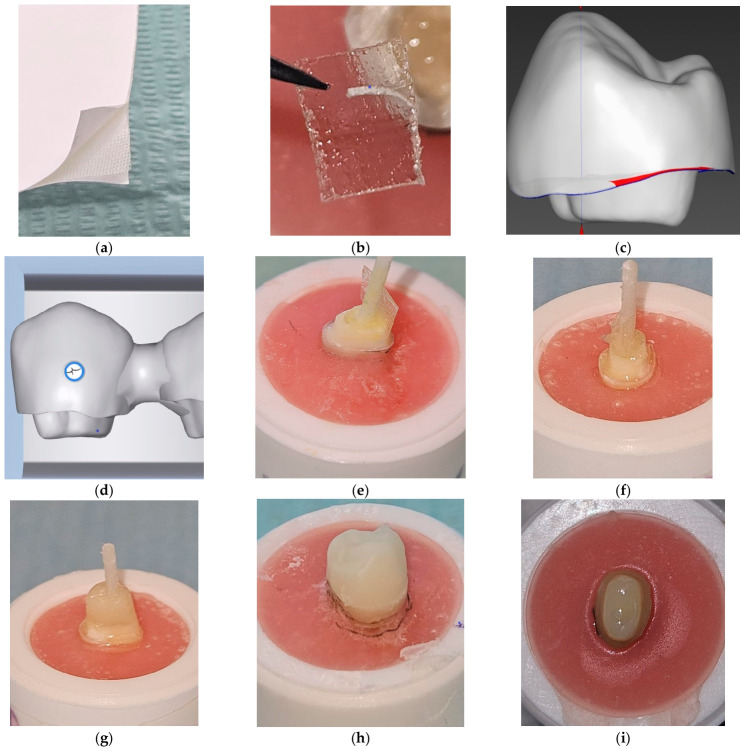
Representative images of the restoration fabrication stages in the FR groups: (**a**) fiber strip material; (**b**) appearance of the fiber strip before application to the EC cavity; (**c**,**d**) CAD stage of the EC restoration; (**e**–**g**) adaptation of the fiber strip and formation of the core structure in the PC restoration; (**h**) pre-polishing inspection of the CAM-fabricated restoration; and (**i**) application of the bonding agent to the tooth surface before cementation in the PC restoration. FR: fiber strip-reinforced; EC: endocrown; PC: post-core; CAD: computer-aided design; CAM: computer-aided manufacturing.

In the NF groups, the cavity surfaces were etched with 37% phosphoric acid for 30 s to ensure micromechanical retention. The acid-treated surfaces were then left undisturbed for 15 s, rinsed with copious water, and gently air-dried. After etching, an adhesive agent (Scotchbond Universal, 3M ESPE, St. Paul, MN, USA) was applied and polymerized using a light-emitting diode (LED) curing unit (Elipar DeepCure, 3M ESPE, Seefeld, Germany) with a minimum intensity of 1470 mW/cm^2^. In the FR groups, flowable resin (G-aenial Universal Injectable, GC Dental, Tokyo, Japan) was first placed at the base of the cavity; two layers of fiber strip were cut with sharp scissors and adapted to the cavity using hand instruments. According to the manufacturer’s information, the GF strip used in this study consisted of silanated E-glass fibers impregnated with bisphenol A-glycidyl methacrylate (Bis-GMA) and poly(methyl methacrylate) (PMMA) and had a nominal thickness of 0.1 mm [30]. In the EC-FR groups, the two strip layers were oriented parallel to the pulpal floor and adapted as a flat reinforcing layer within the base structure. For all EC-FR specimens, the same strip thickness, number of layers, orientation parallel to the pulpal floor, adaptation sequence, and curing protocol were used to improve reproducibility and reduce operator-related variability. Flowable resin was then applied again, and the fiber strip–resin complex was polymerized using an LED curing unit for 40 s. Analysis of the gel content of the polymerized resin matrix within the fiber strip–resin complex was not included in the experimental protocol because polymer matrix characterization was outside the scope of the present FS testing design, and the fiber strip–resin complex contained insoluble inorganic filler and GF components [30,31]. After polymerization, the specimens were stored in distilled water at 4 °C for 24 h (Figure 2).

The EC cavity design was performed according to the dimensions reported in previous studies [32,33,34]. The cavity depth was adjusted to 3 mm using a graduated periodontal probe. The axial wall thickness was standardized to 2 mm. The finish line was prepared in a butt-joint configuration and positioned approximately 2 mm above the CEJ. Final adjustments were made using an extra-fine round-ended diamond bur.

### 2.4. Post Space Preparation in the PC Groups

Teeth were embedded in polypropylene tubes (2.2 cm diameter, 3.5 cm height) with self-polymerizing acrylic resin (ProBase Cold, Ivoclar Vivadent, Schaan, Liechtenstein) using the same protocol as in the EC group. GP removal was performed using Gates-Glidden drills (Dentsply Maillefer, Ballaigues, Switzerland).

The post space was prepared using a post preparation system (RelyX™ Fiber Post kit, 3M ESPE, St. Paul, MN, USA). Root preparation was performed at 2000 rpm with a speed-controlled angle handpiece. The post space was created using a #4 Gates-Glidden drill to a depth of 8 mm from the buccal CEJ. This depth was selected to ensure adequate post retention and minimize the risk of root perforation [35].

A GF post (Cytec HT Glassfiber, Hannenkratt, Königsbach-Stein, Germany; Ø1.4 mm) was placed in the root canal, and its fit was verified. Before cementation, the root canals were cleaned by irrigation with 5.25% NaOCl. The canal walls were etched with 37% phosphoric acid (Etchant, Ivoclar Vivadent, Schaan, Liechtenstein) for 15 s, then rinsed with distilled water and gently air-dried. A universal bonding agent (G-Premio Bond, GC Dental, Tokyo, Japan) was applied to the canal walls and light-cured using an LED curing unit (Elipar DeepCure, 3M ESPE, Seefeld, Germany) for 20 s. The post was inserted into the canal using adhesive resin cement (RelyX™ Unicem Self-Adhesive Resin Cement, 3M ESPE, St. Paul, MN, USA) and light-cured for 40 s.

### 2.5. Core Build-Up and Tooth Preparation in the PC Groups

In the FR groups, a bonding agent (G-Premio Bond, GC Dental, Tokyo, Japan) was applied to the surface of the post. The periphery of the post was then coated with a flowable RC (G-aenial Universal Injectable, GC Dental, Tokyo, Japan). Two layers of fiber strips were circumferentially adapted onto this resin layer and light-cured with an LED unit for 20 s on each surface. This procedure resulted in an additional fiber strip support layer surrounding the coronal region of the post. Subsequently, core structures were constructed using a nanocomposite RC applied with the layering technique (Figure 2). For all PC-FR specimens, the same strip thickness, number of layers, circumferential orientation, adaptation sequence, and curing protocol were used to improve reproducibility and reduce operator-related variability.

In the NF group, the same bonding agent was applied without placing a fiber strip around the post. Then, core structures were created using a layering technique with nanocomposite RC. A core 4.5 mm high was built on the ferrule in 1.5 mm increments; each layer was light-cured separately with LED light. Final adjustments to the core structure were performed using fine diamond finishing burs.

During the preparation phase, a cavity with a 6–8° taper was obtained using a flame-shaped diamond bur. The finishing line was positioned approximately 0.5 mm above the CEJ, and a 1 mm wide chamfer finish line was created circumferentially. A 2 mm high circumferential ferrule was provided around the tooth. After final adjustments, the total core height was set to 5.5 mm to ensure that the occlusal ceramic thickness in the final restoration was at least 1.5 mm according to the manufacturer’s guidelines [36].

### 2.6. Fabrication and Cementation of Restorations

EC and PC restorations were fabricated using CAD/CAM technology. Specimens were recorded in three dimensions using an intraoral scanner (CEREC Omnicam 4.5, Dentsply Sirona, Bensheim, Germany). All restoration types were created using appropriate CAD software (CEREC InLab SW 22.1, Dentsply Sirona, Bensheim, Germany) and standardized to a cement gap of 80 µm [24]. The biogeneric design mode of the software was used during the design process. Milling of the restorations was performed using a CAM device (CEREC MC XL, Dentsply Sirona, Germany). After milling, the specimens were polished using a polishing kit (EVE Flexi-Dia, Ernst Vetter GmbH, Keltern, Germany) according to the manufacturer’s instructions. The crown length was set at approximately 7 mm based on the average anatomical dimensions of mandibular premolar teeth [37].

Before cementation, the intaglio surfaces of the restorations were prepared according to the material type and the manufacturers’ surface pretreatment recommendations [36,38]. For FC restorations, the intaglio surfaces were etched with 5% hydrofluoric acid for 60 s, thoroughly rinsed with water, air-dried, and treated with a silane coupling agent (G-Multi PRIMER, GC Dental, Tokyo, Japan). For RNC restorations, the intaglio surfaces were etched with 5% hydrofluoric acid for 60 s according to the hydrofluoric acid etching protocol recommended by the manufacturer, thoroughly rinsed with water spray, dried, and treated with the same silane coupling agent.

The tooth preparation surfaces were etched with 37% phosphoric acid for 15 s, rinsed with distilled water, and gently air-dried. A single-component universal adhesive agent (G-Premio Bond, GC Dental, Tokyo, Japan) was then applied according to the manufacturer’s instructions and light-cured for 20 s using an LED curing unit. After these surface pretreatment procedures, all restorations were cemented using the same dual-cure resin cement (G-CEM One, GC Dental, Tokyo, Japan) following a standardized cementation protocol. During cementation, a constant load of 5 kg was applied to the restorations using a cementation apparatus and maintained for 60 s [39]. Each surface was light-cured for 40 s using an LED curing unit. Excess cement was removed using fine-tipped hand instruments and fine-grained burs. The crown margins adjusted with burs were polished again using the same polishing kit.

### 2.7. Thermal Aging

All specimens were aged in a thermocycler (SD Mechatronik Thermocycler, Feldkirchen-Westerham, Germany) to simulate intraoral conditions. During the thermocycling process, the specimens were subjected to a total of 10,000 cycles, alternating between temperatures of 5 °C and 55 °C, in accordance with the ISO/TS 11405:2015 standard [40]. The dwell time in each bath was set to 30 s, and the transfer time between baths was set to 10 s. This aging protocol was considered to simulate 1 year of clinical use [41]. Following the aging process, the specimens were stored in distilled water at 4 °C until mechanical testing was performed.

### 2.8. FS Test

Following the aging process, all specimens were subjected to FS testing using a universal testing machine (Lloyd LRX, Lloyd Instruments, Fareham, UK). The steel ball tip (Ø = 4 mm) used for loading was positioned on the buccal crest of the buccal cusp of the mandibular premolar, parallel to the long axis of the tooth, to simulate a clinically relevant occlusal loading point [42]. The load was applied as a compressive force at a crosshead velocity of 0.5 mm/min, and the maximum force at fracture was recorded in Newtons (N). After the fracture, the specimens were examined to determine the FP of each specimen.

### 2.9. Evaluation of FPs

All specimens were examined under a stereomicroscope (Leica MZ12, Leica Microsystems, Wetzlar, Switzerland) at 8× magnification. FPs were classified as follows, in line with previous studies [43]: Type I, separation of EC or PC without fracture; Type II, fracture of EC or PC without involvement of the tooth structure; Type III, fracture involving tooth structure above the CEJ level; and Type IV, fracture involving tooth structure at or below the CEJ level. Types I, II, and III were considered repairable, whereas Type IV was classified as an irreparable (catastrophic) failure. Crack propagation values, such as crack length or propagation rate, were not quantitatively measured; stereomicroscopic evaluation was used only for FP classification.

### 2.10. Statistical Analysis

Statistical analysis of FS data was performed using statistical software (IBM SPSS Statistics, Version 23.0, IBM Corp., Armonk, NY, USA). The normality of the data distribution was assessed using the Shapiro–Wilk test, and the FS data were found to be normally distributed. Homogeneity of variances was confirmed using the Levene test.

To evaluate the main effects and interactions on FS, a three-way analysis of variance (ANOVA) was applied, including the factors of material type (RNC, FC), restoration type (PC, EC), and reinforcement (NF, FR). Bonferroni-corrected post hoc tests were used for comparisons where significant differences were found. Effect sizes were reported using partial η^2^ values. The significance level was set at α = 0.05 for all statistical analyses. The 95% confidence intervals for the interaction means were calculated consistently from the three-way ANOVA model using the pooled error term and were rounded to the nearest whole Newton (N).

## 3. Results

The results of the three-way ANOVA regarding the effects of material, restoration type, and reinforcement on FS are presented in Table 2. Accordingly, the main effects of material, restoration type, and reinforcement, as well as the material × restoration, restoration × reinforcement, and material × restoration × reinforcement interactions, were found to be statistically significant for FS (*p* < 0.05).

When evaluated independently of restoration type and reinforcement, the FS value of RNC (861 ± 181 N) was higher than that of FC (715 ± 212 N) (*p* < 0.001) (Table 3).

When restoration type was evaluated independently of material and reinforcement, the FS value of EC (828 ± 173 N) was higher than that of PC (748 ± 236 N) (*p* = 0.046).

When reinforcement was evaluated independently of material and restoration type, the FS value of FR (848 ± 180 N) was higher than that of NF (728 ± 222 N) (*p* = 0.003).

When the material–restoration interaction was evaluated independently of reinforcement, FS values were ranked from highest to lowest as follows: RNC–PC (883 ± 191 N) > RNC–EC (840 ± 173 N) > FC–EC (816 ± 178 N) > FC–PC (613 ± 198 N) (*p* = 0.003) (Table 4).

When the material–reinforcement interaction was evaluated independently of restoration type, FS values differed among the RNC–FR (937 ± 150 N), RNC–NF (785 ± 181 N), FC–FR (759 ± 165 N), and FC–NF (671 ± 248 N) combinations; however, these differences were not statistically significant (*p* > 0.05). This indicates that, when EC and PC restorations were pooled, the effect of fiber strip reinforcement on FS did not differ significantly between RNC and FC materials.

When the restoration–reinforcement interaction was evaluated independently of material, FS values were ranked from highest to lowest as follows: PC–FR (856 ± 167 N) > EC–FR (840 ± 196 N) > EC–NF (816 ± 152 N) > PC–NF (640 ± 249 N). This interaction was statistically significant for FS (*p* = 0.018).

When the effect of the material–restoration–reinforcement three-way interaction on FS was evaluated, the highest FS value was observed in the RNC–PC–FR group (965 ± 144 N), whereas the lowest value was observed in the FC–PC–NF group (480 ± 177 N) (*p* = 0.039). Based on the mean FS values, significant differences were observed among the material–restoration–reinforcement combinations (Table 3).

When all specimens were considered together (N = 72), Type IV FP was the most frequently observed, accounting for 35% (*n* = 25). This was followed by Type III (33%; *n* = 24) and Type II (29%; *n* = 21). Type I FP was rarely observed, constituting 3% (*n* = 2).

**Figure 3:**
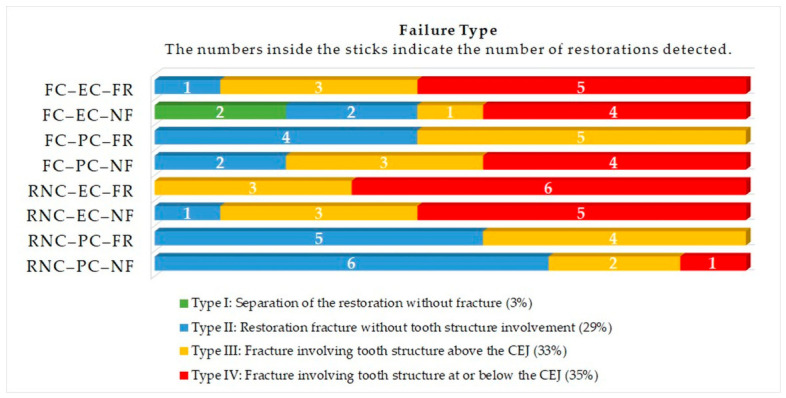
Distribution of FPs among the tested groups. FC, feldspathic ceramic; RNC, resin-nanoceramic; EC, endocrown; PC, post-core; NF, fiber strip-non-reinforced; FR, fiber strip-reinforced; CEJ, cementoenamel junction.

When evaluated according to the experimental groups, the highest number of Type IV FPs was observed in the RNC–EC–FR group. In contrast, the RNC–PC–NF group showed the highest frequency of Type II failures. Overall, restorations fabricated from RNC tended to exhibit slightly higher proportions of repairable failure patterns (Type I–III) compared with FC groups. The distribution of FPs across the experimental groups is presented in Figure 3.

**Figure 4:**
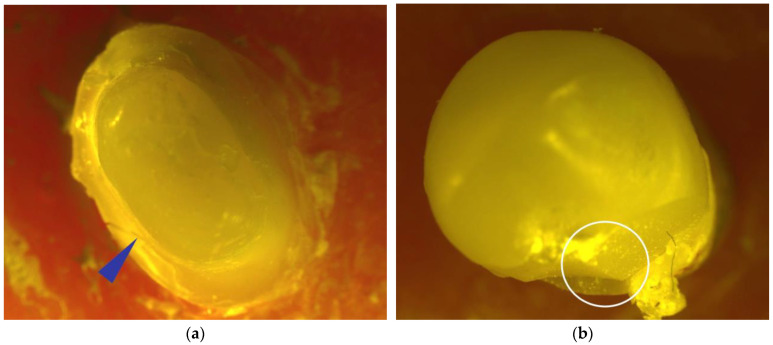

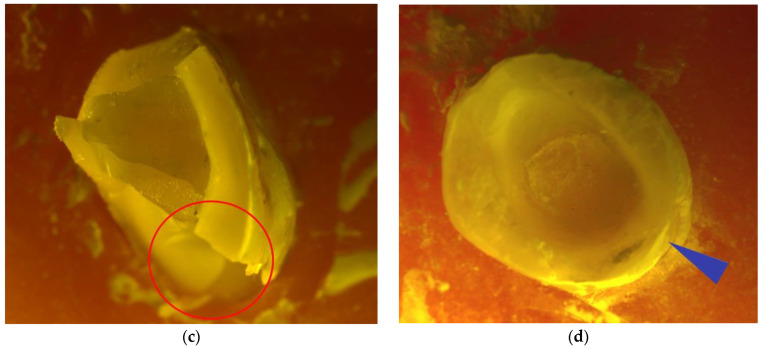
Representative stereomicroscope images of teeth restored with RNC: (**a**) PC-NF specimen, (**b**) PC-FR specimen, (**c**) EC-NF specimen, and (**d**) EC-FR specimen. FC, feldspathic ceramic; RNC, resin-nanoceramic; EC, endocrown; PC, post-core; NF, fiber strip-non-reinforced; FR, fiber strip-reinforced. Blue arrows indicate fracture lines, white circles indicate ceramic fragment detachment, and red circles indicate tooth tissue loss.

Figure 4 shows representative stereomicroscope images obtained after fracture testing in teeth restored with RNC. Figure 4a presents a stereomicroscope image of the RNC-PC-NF specimen. The fracture line was concentrated at the crown and coronal dentin level; the apical continuity of the post was maintained, and it did not extend into the radicular dentin. This FP was classified as Type III. In Figure 4b, only a restoration fracture was detected in the RNC-PC-FR specimen; a distinct fracture line was not observed in the dentin or core structure. This FP was evaluated as Type II. In Figure 4c, the fracture line in the RNC–EC–NF specimen was observed to extend along the pulp chamber walls below the CEJ, indicating radicular dentin involvement. This FP was evaluated as Type IV. In Figure 4d, the EC restoration in the RNC-EC-FR specimen was associated with a fracture involving the tooth structure above the CEJ, without extension into the radicular dentin. This FP was classified as Type III.

**Figure 5:**
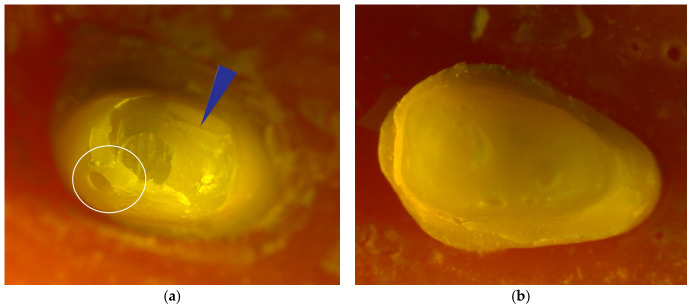

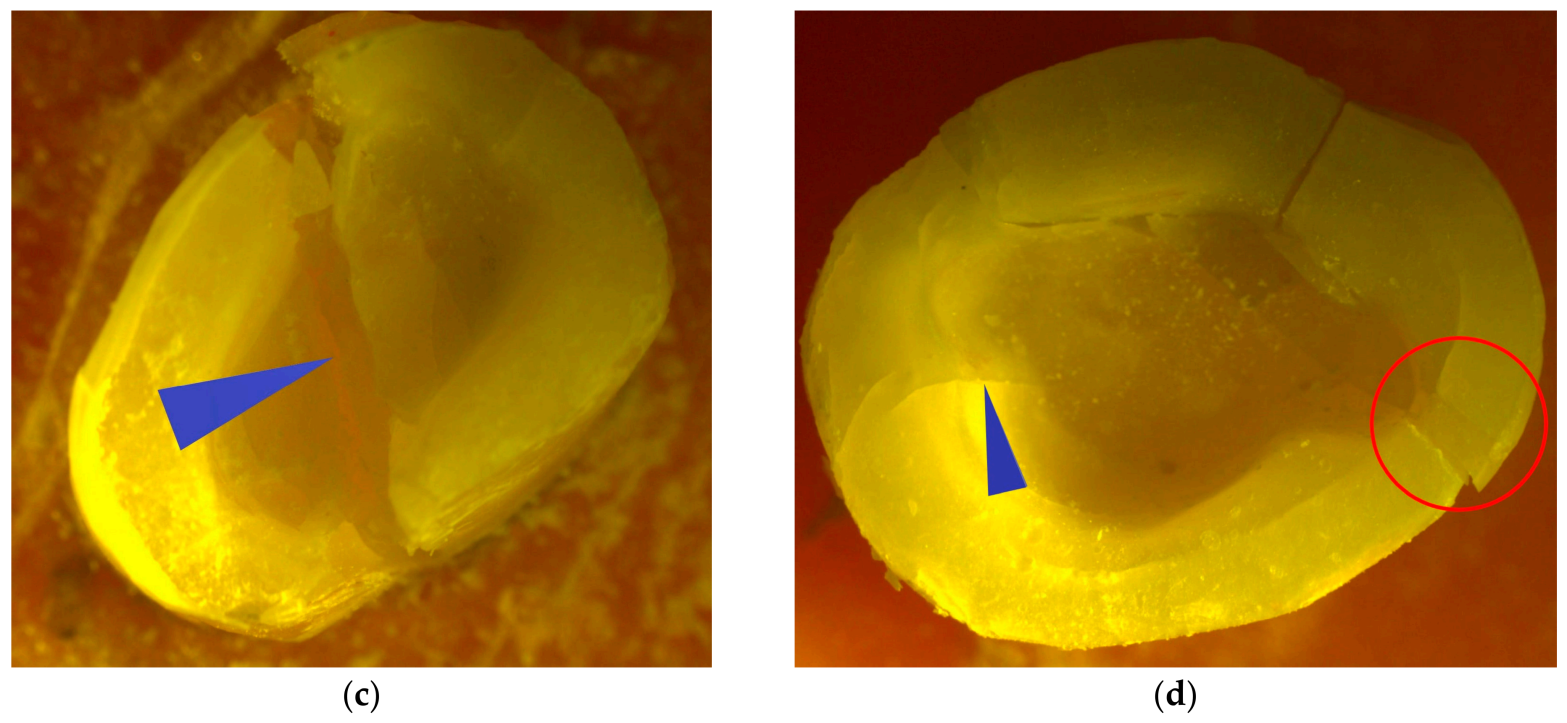
Representative stereomicroscope images of teeth restored with FC: (**a**) PC-NF specimen, (**b**) PC-FR specimen, (**c**) EC-NF specimen, and (**d**) EC-FR specimen. FC, feldspathic ceramic; RNC, resin-nanoceramic; EC, endocrown; PC, post-core; NF, fiber strip-non-reinforced; FR, fiber strip-reinforced. Blue arrows indicate fracture lines, white circles indicate ceramic fragment detachment, and red circles indicate tooth tissue loss.

In Figure 5a, a fracture was observed in the coronal portion of the GF post in the FC-PC-NF specimen while maintaining apical continuity, and the fracture was confined to the crown–coronal dentin. This FP was classified as Type III. In Figure 5b, only a restoration fracture was detected in the FC-PC-FR specimen, and this FP was classified as Type II. In Figure 5c, the FP in the FC–EC–NF specimen progressed along the pulp chamber and extended below the CEJ, involving the cervical tooth structure, and was evaluated as Type IV. In Figure 5d, the fracture line in the FC-EC-FR specimen was observed to be concentrated around the pulp chamber and did not extend to the radicular dentin; this FP was classified as Type III.

## 4. Discussion

The null hypothesis was rejected, as material, restoration type, and reinforcement significantly affected FS. Among the tested combinations, the highest FS value was observed in the RNC–PC–FR group, whereas the lowest FS value was observed in the FC–PC–NF group. In the material × restoration type interaction, the FC–PC combination showed lower FS values than the other material–restoration type combinations (*p* = 0.003). In the restoration type × reinforcement interaction, the FS-enhancing effect of fiber strip reinforcement was more pronounced in PC restorations than in EC restorations (*p* = 0.018). These findings indicate that FS varied according to group combinations rather than individual factors alone.

In vitro models are widely used to evaluate the mechanical performance of restorative materials under standardized conditions and to compare findings with previously reported FS ranges [44]. Because controlled fracture tests cannot be ethically or methodologically performed in human subjects, such evaluations are commonly conducted under laboratory conditions [45]. Mandibular premolars provide a suitable experimental model for in vitro studies because they are generally single-rooted, have a morphology that can be more easily standardized, and represent a clinically relevant tooth group in posterior restorative biomechanics [12]. Therefore, consistent with previous studies [8,9,46,47], the present study was designed as an in vitro investigation using mandibular premolar teeth.

In this study, natural teeth were preferred over artificial teeth to better reflect the E values of dentin and enamel and the thin, conical root morphology of premolars. The use of natural teeth allowed the FP of the restoration–tooth complex to be evaluated on natural tooth structures [22]. Periodontal ligament simulation was not performed to avoid additional variability, because materials used to mimic the periodontal ligament may deform or degrade under mechanical testing conditions, and variations in layer thickness can influence load transfer and stress distribution [48].

In fracture testing studies, axial loading has been reported to provide more controlled and reproducible load transmission than oblique loading by reducing lateral and bending moment components [49]. In contrast, studies using oblique loading angles such as 45° [12,46], 30° [50,51], and 20° [47] have reported lower fracture loads and more complex FPs due to the influence of lateral forces and bending moments. Therefore, caution should be exercised when directly comparing results obtained under different loading angles, and the findings should be interpreted within their specific biomechanical testing conditions [52]. In the present study, fracture testing was performed under 90° axial loading, consistent with previous studies [8,53,54], to enhance experimental standardization rather than to fully reproduce multidirectional clinical occlusal loading.

Previous studies [55,56] have reported that normal functional masticatory forces in the premolar region are approximately 520 N. Another study [22] used 750 N as a threshold for interpreting the mechanical performance of restorative systems in premolars under high-load conditions. Therefore, in the present study, the 520 N and 750 N values were used as interpretive thresholds when evaluating the FS results obtained under the present in vitro conditions.

When the tested materials were evaluated independently of reinforcement and restoration type, the RNC group exceeded both the 520 N functional threshold and the 750 N high-load threshold, with a mean FS value of 861 N. In contrast, the FC group exceeded the 520 N functional threshold, with a mean FS value of 715 N; however, it remained below the 750 N high-load threshold. This difference indicates a greater FS margin and therefore a more favorable in vitro mechanical performance for RNC under the present standardized loading conditions. Clinical survival, however, represents the long-term maintenance of the restoration in the oral environment, where outcomes are determined not only by FS but also by fatigue behavior, bonding durability, marginal integrity, biological factors, occlusal loading, and patient-related variables. Therefore, the present findings should be interpreted as mechanical indicators that may guide material-, restoration-, and reinforcement-related decisions for improving potential clinical performance, rather than as direct evidence of long-term clinical survival.

The E of the restorative material is an important parameter influencing the biomechanical behavior of restorative systems [57]. In the present study, the higher mean FS observed for RNC than for FC may be partly explained by the closer match between the reported E value of RNC and that of dentin. The RNC material used in this study has a reported E value of 9.6 GPa [58], whereas the FC material has been reported to have a substantially higher E value of approximately 45 GPa [36]. Dentin has been reported to exhibit E values ranging from approximately 12 to 25 GPa, depending on the testing method [59]. This modulus compatibility may contribute to a more favorable biomechanical response by reducing the stiffness mismatch within the restoration–tooth complex. In addition, CAD/CAM resin-matrix ceramic materials are characterized by an organic/polymer matrix highly filled with ceramic particles, and they have been reported to more closely simulate the E of dentin than traditional ceramics [60,61,62]. In contrast, the higher stiffness of FC may promote localized stress concentration under load, potentially creating regions susceptible to crack initiation [63].

One of the important factors affecting the FS of the restoration is the amount of remaining tooth structure in the restored tooth [64]. Atmeh et al. [8] reported that the amount of remaining coronal dentin and the cavity volume are decisive factors in FS, and that FS decreases significantly as dentin loss increases. Therefore, preparation was standardized in all specimens to control the effect of the amount of remaining tooth structure on FS.

Previous studies [51,65] have reported that limiting the pulp chamber depth to 2–4 mm in EC restorations applied to posterior teeth provides sufficient biomechanical performance and that deep preparations extending to the root canal dentin should be avoided. Accordingly, the cavity depth for EC restorations was set at 3 mm in the present study. Consistent with previous studies [8,51,53], the minimum wall thickness was standardized to 2 mm for both EC and PC restorations, and a 2 mm circumferential ferrule extending coronally from the CEJ was created for PC restorations.

Manufacturer guidelines were followed in CAD/CAM restoration production, and software and production parameters were kept constant for all groups, considering that restoration geometry and design parameters may affect FS [36,51]. Geometric variation was minimized by initially selecting teeth with similar size and morphology. Cusp inclinations and buccolingual cusp proportions were adjusted within the limits of the biogeneric algorithm to obtain a standardized restoration design.

For EC restorations, the occlusal anatomy and overall restoration geometry were standardized in accordance with previous studies [66,67]. Since cusp morphology has been reported to affect fracture loads [8], the occlusal geometry was designed according to the same principles in all models.

A previous study [68] reported that the amount of remaining tooth structure is decisive for restoration strength and that the presence of a ferrule increases FS, especially in PC systems. In PC restorations, load transfer is directed to the radicular dentin via the ferrule and post, whereas EC restorations are defined as conservative designs that provide retention from the pulp chamber and do not require a peripheral ferrule [6]. Therefore, evaluating EC and PC designs under FR and NF conditions allowed comparison of the effect of fiber strip reinforcement on FS in restorations with different structural designs and load transfer patterns.

When fiber strip reinforcement was evaluated independently of material and restoration type, the FR groups exhibited higher mean FS than the NF groups (848 N vs. 728 N), consistent with previous studies [8,53]. This increase may be related to the ability of fibers to modify stress transfer under load and to deflect crack propagation within the resin matrix [53]. Fibers may also bridge crack tips and thereby contribute to increased fracture toughness [8]. In the present study, two layers of fiber strips were applied as an intermediate reinforcing network within the resin matrix to ensure adequate adaptation to the base/core structure [24]. This bidirectional fiber network may have contributed to the higher FS observed in the FR groups by supporting stress redistribution and crack deflection.

The type and configuration of the GF strips should also be considered when interpreting the reinforcing effect. In the present study, pre-impregnated bidirectional GF strips were used, and two layers were incorporated into the resin matrix. Previous reports [69,70] have emphasized that the reinforcing effect of GF-reinforced composites depends on fiber composition, orientation, distribution, amount, length, and adhesion to the resin matrix. Bidirectional fiber configurations may support stress redistribution in more than one direction, whereas the position of the fiber layer may influence crack deflection and the resulting FPs. Consistently, Saratti et al. [71] reported that the presence and position of bidirectional E-glass fibers beneath a CAD/CAM RC influenced fracture behavior, with fiber-containing groups showing horizontal crack deflection and a toughening effect after static and fatigue loading. Therefore, the FS and FP findings of the present study should be interpreted in relation to the bidirectional structure, two-layer application, and base/core position of the GF strips.

In EC restorations, which represent a ferrule-free restorative design, the contribution of fiber strip reinforcement appeared more limited than in PC restorations. A previous study [72] reported that fiber reinforcement may contribute to the mechanical stability of teeth restored without a ferrule. In the present study, fiber strip reinforcement was associated with a numerical increase in FS in EC restorations fabricated from RNC, whereas it did not increase FS in EC restorations fabricated from FC. Finite element analysis-based studies [73,74] have suggested that fiber placement in restorations with limited intracoronal cavity volume may lead to localized stress concentrations at the cavity floor and surrounding regions, which could reduce the reinforcing effect of fibers, particularly in relatively stiff ceramic materials. In contrast, the more compliant mechanical behavior of RNC materials may allow partial stress dissipation, as reported previously [75,76], and may help explain the more favorable FS response observed in the RNC–EC–FR group. Regardless of fiber strip reinforcement, the mean FS values of EC restorations remained above the reported functional load threshold under the present in vitro conditions.

In PC restorations, load transfer occurs through the ferrule, post, and radicular dentin, and this structural configuration may influence the contribution of fiber strip reinforcement depending on the restorative material. In the present study, PC restorations fabricated from FC showed lower FS values than those fabricated from RNC. Fiber strip reinforcement was associated with increased FS, particularly in FC–PC restorations. Zicari et al. [77] showed that the ferrule effect significantly enhanced the fracture resistance of endodontically treated teeth after fatigue loading. Kharouf et al. [78] reported that cavity configuration and post-endodontic restoration type influenced FS, FPs, and stress distribution in premolars. In this context, the GF strip may have acted as a stress-modulating layer around the coronal post/core region, especially when combined with the relatively stiff FC restoration. In contrast, RNC–PC restorations remained above the reported high-load threshold regardless of fiber strip reinforcement. These findings suggest that fiber strip reinforcement may have greater mechanical relevance in PC restorations fabricated from relatively stiff and brittle ceramic materials, whereas its additional effect may be less apparent in RNC–PC restorations because their mean FS values were already high under the present in vitro conditions.

In EC restorations, all FR and NF combinations in both RNC and FC groups exceeded the 750 N high-load threshold under the present in vitro conditions. This finding indicates that the mean FS values of EC restorations remained above the reported high-load threshold across the tested material and reinforcement conditions. However, the effect of fiber strip reinforcement varied according to the restorative material, increasing FS in RNC–EC restorations but not in FC–EC restorations. Therefore, fiber strip reinforcement should not be interpreted as a universal reinforcement strategy; rather, its contribution appears to depend on the specific material–restoration–reinforcement combination.

The evaluation of FPs after FS testing provided additional information on the fracture behavior of the restoration–tooth complex. Ahmed et al. [51] reported that fracture lines in EC restorations may initiate in the cervical region and at the pulp chamber floor, and that stress concentrations in these regions may contribute to fracture formation when they are not sufficiently dissipated within the restorative material and are transferred to dentin. It has also been suggested that the thin and conical root morphology of premolar teeth may facilitate stress transmission and promote faster apico–coronal fracture propagation. In the present study, Type IV fractures were more frequently observed in EC restorations than in PC restorations. This distribution showed a trend consistent with the proposed biomechanical mechanism.

It should also be noted that higher FS values do not necessarily indicate more repairable FPs. Amin et al. [79] reported that, in endodontically treated premolars, full-coverage restorations supported by a PC design showed more repairable FPs than some partial-coverage alternatives. Al Fodeh et al. [80] also evaluated premolar EC restorations in terms of both FS and FPs and showed that preparation design and restorative material can affect not only FS but also FPs. Therefore, FS findings should be interpreted together with FP distribution, particularly in premolar EC restorations, because cervical dentin thickness, pulp chamber geometry, and ferrule-free load transfer may influence the risk of root-involving fractures.

The butt-joint preparation used for EC restorations provides a conservative design; however, the limited coronal dentin coverage around the cervical region may have contributed to stress concentration around the cervical and pulp chamber areas under axial loading. This may be one possible explanation for the more frequent Type IV fractures observed in EC restorations. In contrast, the ferrule-supported preparation design in PC restorations may have provided an additional circumferential resistance form and helped maintain coronal dentin continuity, which could be associated with the lower frequency of root-involving fractures observed in these groups.

When the restoration type × reinforcement interaction was considered in relation to FP distribution, Type III fractures were more frequently observed in PC–FR restorations than in PC–NF restorations. In addition, no Type IV fractures were observed in the PC–FR groups, whereas Type IV fractures were observed in the PC–NF groups. This distribution is consistent with previous biomechanical studies [8,53], suggesting that fiber strip reinforcement may contribute to more repairable FPs by supporting stress redistribution and crack deflection.

Type II and Type III FPs were more frequently observed in PC restorations than in EC restorations. This distribution may be related to the ferrule-supported design and the use of GF posts, whose E is closer to that of dentin. These features may support more compatible load transfer between the post, core, and radicular dentin and may help limit fracture progression into the radicular dentin. This may explain the more frequent observation of repairable FPs in PC restorations under the present in vitro conditions.

The present study has several limitations. FS tests were performed under 90° axial static loading, and dynamic, oblique, or lateral loading conditions were not applied; therefore, the complex loading characteristics of clinical chewing conditions were not fully simulated. Fatigue loading was not performed; therefore, the cumulative effects of repeated masticatory loading could not be evaluated. Bond strength was not separately evaluated; therefore, the specific contribution of adhesive interface strength to the FS and FP outcomes could not be directly determined. Furthermore, periodontal ligament simulation was not performed, which may have influenced load transfer and fracture behavior under the present experimental conditions. The gel content and degree of conversion of the polymerized resin matrix within the fiber strip–resin complex were not measured. Because this complex consisted of a filled flowable RC and GF strips, direct gel content analysis would not specifically isolate resin matrix crosslinking. Therefore, although these parameters may influence the mechanical behavior of resin-based restorative materials [81,82], their specific contribution to the FS and FP outcomes of the reinforced groups could not be determined. Although the use of natural premolar teeth contributed to biological realism, individual variations related to dentin quality and root morphology could not be completely eliminated. Furthermore, FPs were evaluated qualitatively under a stereomicroscope, and advanced imaging or quantitative deformation analysis methods were not applied. Further studies are needed to investigate the biomechanical effects of fiber strip reinforcement in CAD/CAM restorative systems under different restoration designs and loading conditions.

## 5. Conclusions

Within the limitations of this in vitro study, FS was affected by restorative material, restoration type, and fiber strip reinforcement. Overall, RNC restorations showed higher mean FS than FC restorations (861 ± 181 N vs. 715 ± 212 N), and FR groups showed higher mean FS than NF groups (848 ± 180 N vs. 728 ± 222 N). However, these effects were not uniform across all combinations of material, restoration type, and reinforcement. Although EC restorations showed higher mean FS than PC restorations at the main-effect level (828 ± 173 N vs. 748 ± 236 N), the effect of restoration type depended on the restorative material and reinforcement condition. The highest FS was observed in the RNC–PC–FR group (965 ± 144 N), whereas the lowest FS was found in the FC–PC–NF group (480 ± 177 N). In the FP distribution, FR tended to be associated with more repairable FPs, particularly in PC restorations.

From a clinical perspective, the results may support a material- and design-specific approach to the restoration of structurally compromised endodontically treated premolars. Fiber strip reinforcement was associated with a more consistent increase in FS in ferrule-supported PC restorations with both materials, whereas its effect in butt-joint EC restorations appeared to be material-dependent and was mainly evident in the RNC–EC group. These findings may contribute to optimizing current clinical protocols by guiding reinforcement decisions according to restorative material and restoration design.

## Figures and Tables

**Table 1:** Composition of the materials used in the study.

Material	Use in the Study	Material Type	Composition	Manufacturer
Cerec Blocs C	FC restorative material	Industrially manufactured fine-structured FC	Oxide composition by weight: SiO_2_ 56–64%, Al_2_O_3_ 20–23%, Na_2_O 6–9%, K_2_O 6–8%, CaO 0.3–0.8%, TiO_2_ 0.0–0.1%, and pigments < 0.1%.	Dentsply Sirona, Bensheim, Germany
Cerasmart 270	RNC restorative material	Hybrid ceramic block containing ultrafine homogeneously dispersed fillers in a highly cross-linked resin matrix	Inorganic phase: 71 wt%, including silica nanoparticles, barium glass ceramic particles, strontium- and aluminum-containing glass phase, and nanoceramic fillers. Organic phase: 29 wt%, including UDMA, Bis-MEPP, DMA, and auxiliary dimethacrylates. Filler particle sizes are approximately 20 nm for silica and 300 nm for barium glass.	GC Corp., Tokyo, Japan
Filtek Ultimate	Nanocomposite RC for core build-up in PC groups	Visible-light-activated nanocomposite RC	Resin system: bis-GMA, UDMA, TEGDMA, bis-EMA(6), and PEGDMA. Filler system: 20 nm silica, 4–11 nm zirconia, and zirconia/silica clusters. Cluster particle size is 0.6–10 µm for DEB shades and 0.6–20 µm for translucent shades. Filler loading is 78.5 wt%/63.3 vol% for non-translucent shades and 72.5 wt%/55.6 vol% for translucent shades.	3M ESPE, Seefeld, Germany
G-aenial Universal Injectable	Flowable RC for fiber strip adaptation in the EC-FR and PC-FR groups	Light-cured, radiopaque injectable RC	Matrix: methacrylate monomer, 31 wt%. Fillers: silica and barium glass, 69 wt%, including approximately 150 nm ultrafine barium fillers with full-coverage silane coating technology. Pigments and photoinitiators are present in trace amounts.	GC Dental, Tokyo, Japan

FC, feldspathic ceramic; RNC, resin nanoceramic; RC, resin composite; PC, post-core; EC-FR, fiber strip-reinforced endocrown; PC-FR, fiber strip-reinforced post-core; DEB, dentin, enamel, and body; UDMA, urethane dimethacrylate; Bis-MEPP, bisphenol A ethoxylate dimethacrylate; DMA, dimethacrylate; bis-GMA, bisphenol A-glycidyl methacrylate; TEGDMA, triethylene glycol dimethacrylate; bis-EMA(6), bisphenol A polyethylene glycol diether dimethacrylate; PEGDMA, polyethylene glycol dimethacrylate; SiO_2_, silicon dioxide; Al_2_O_3_, aluminum oxide; Na_2_O, sodium oxide; K_2_O, potassium oxide; CaO, calcium oxide; TiO_2_, titanium dioxide; wt%, weight percentage; vol%, volume percentage.

**Table 2:** Three-way ANOVA results for FS.

Source	Sum of Squares	df	F	*p*-Value	Partial η^2^
Material	386,205.140	1	13.930	**<0.001**	0.179
Restoration	115,101.021	1	4.151	**0.046**	0.061
Reinforcement	259,186.500	1	9.348	**0.003**	0.127
Material × restoration	271,865.554	1	9.806	**0.003**	0.133
Material × reinforcement	18,356.478	1	0.662	0.419	0.010
Restoration × reinforcement	163,702.844	1	5.904	**0.018**	0.084
Material × restoration × reinforcement	123,433.067	1	4.452	**0.039**	0.065

Statistically significant effects are shown in bold (*p* < 0.05).

**Table 3:** Mean FS values (N) ± SD.

Material	Restoration	Reinforcement	Mean ± SD
RNC	PC	NF	801 ± 205
		FR	965 ± 144
		Total	883 ± 191
	EC	NF	770 ± 165
		FR	910 ± 159
		Total	840 ± 173
	Total	NF	785 ± 181
		FR	937 ± 150
		Total	861 ± 181
FC	PC	NF	480 ± 177
		FR	747 ± 110
		Total	613 ± 198
	EC	NF	861 ± 132
		FR	771 ± 213
		Total	816 ± 178
	Total	NF	671 ± 248
		FR	759 ± 165
		Total	715 ± 212
Total	PC	NF	640 ± 249
		FR	856 ± 167
		Total	748 ± 236
	EC	NF	816 ± 152
		FR	840 ± 196
		Total	828 ± 173
	Total	NF	728 ± 222
		FR	848 ± 180
		Total	788 ± 209

FC, feldspathic ceramic; RNC, resin-nanoceramic; EC, endocrown; PC, post-core; NF, fiber strip-non-reinforced; FR, fiber strip-reinforced; SD, standard deviation.

**Table 4:** Results of two-way interactions for FS (N).

Material × Restoration			
Material	Restoration	Mean	95% CI (Lower–Upper)
RNC	PC	883	804–961
	EC	840	761–918
FC	PC	613	535–691
	EC	816	738–894
Material × reinforcement			
Material	Reinforcement	Mean	95% CI (Lower–Upper)
RNC	NF	785	707–863
	FR	937	859–1015
FC	NF	671	592–749
	FR	759	681–837
Restoration × reinforcement			
Restoration	Reinforcement	Mean	95% CI (Lower–Upper)
PC	NF	640	562–719
	FR	856	777–934
EC	NF	816	737–894
	FR	840	762–919

FC, feldspathic ceramic; RNC, resin-nanoceramic; EC, endocrown; PC, post-core; NF, fiber strip-non-reinforced; FR, fiber strip-reinforced; CI, confidence interval.

## Data Availability

The original contributions presented in this study are included in the article. Further inquiries can be directed to the corresponding author.

## Abbreviations

The following abbreviations are used in this manuscript:

ANOVA	Analysis of variance
Bis-GMA	Bisphenol A-glycidyl methacrylate
CAD/CAM	Computer-aided design and computer-aided manufacturing
CEJ	Cementoenamel junction
EC	Endocrown
EDTA	Ethylenediaminetetraacetic acid
ETT	Endodontically treated teeth
FC	Feldspathic ceramic
FP	Fracture pattern
FR	Fiber strip-reinforced
FS	Fracture strength
GF	Glass fiber
GP	Gutta-percha
LED	Light-emitting diode
NaOCl	Sodium hypochlorite
NF	Non-reinforced
PC	Post-core
PMMA	Poly(methyl methacrylate)
RC	Resin composite
RNC	Resin-nanoceramic
